# Reappearance of Minority K103N HIV-1 Variants after Interruption of ART Initiated during Primary HIV-1 Infection

**DOI:** 10.1371/journal.pone.0021734

**Published:** 2011-07-06

**Authors:** Karin J. Metzner, Christine Leemann, Francesca Di Giallonardo, Christina Grube, Alexandra U. Scherrer, Dominique Braun, Herbert Kuster, Rainer Weber, Huldrych F. Guenthard

**Affiliations:** Division of Infectious Diseases and Hospital Epidemiology, University Hospital Zurich, University of Zurich, Zurich, Switzerland; University of Toronto, Canada

## Abstract

**Background:**

In the Zurich Primary HIV infection study (ZPHI), minority drug-resistant HIV-1 variants were detected in some acutely HIV-1-infected patients prior to initiation of early antiretroviral therapy (ART). Here, we investigated the reappearance of minority K103N and M184V HIV-1 variants in these patients who interrupted efficient early ART after 8–27 months according to the study protocol. These mutations are key mutations conferring drug resistance to reverse transcriptase inhibitors and they belong to the most commonly transmitted drug resistance mutations.

**Methodology/Principal Findings:**

Early ART was offered to acutely HIV-1-infected patients enrolled in the longitudinal prospective ZPHI study. Six patients harboring and eleven patients not harboring drug-resistant viruses at low frequencies prior to ART were included in this substudy. Minority K103N and M184V HIV-1 variants were quantified in longitudinal plasma samples after treatment interruption by allele-specific real-time PCR. All 17 patients were infected with HIV-1 subtype B between 04/2003 and 09/2005 and received LPV/r+AZT+3TC during primary HIV-1 infection (PHI). Minority K103N HIV-1 variants reappeared after cessation of ART in two of four patients harboring this variant during PHI and even persisted in one of those patients at frequencies similar to the frequency observed prior to ART (<1%). The K103N mutation did not appear during treatment interruption in any other patient. Minority M184V HIV-1 variants were detected in two patients after ART interruption, one harboring and one not harboring these variants prior to ART.

**Conclusion:**

Minority K103N HIV-1 variants, present in acutely HIV-1 infected patients prior to early ART, can reappear and persist after interruption of suppressive ART containing two nucleoside/nucleotide analogue reverse transcriptase inhibitors and a ritonavir-boosted protease inhibitor.

**Trial Registration:**

Clinicaltrials.gov NCT00537966

## Introduction

Antiretroviral therapy (ART) inhibits human immunodeficiency virus type 1 (HIV-1) in most treated HIV-1-infected patients [1], but eradication of the virus is currently not possible [2], [3], [4]. Virological failure is not uncommon due to the development of drug-resistant viruses [5]. These viruses can be transmitted and can cause therapy failure in treatment-naïve patients [6], [7]. It is therefore recommended to perform drug resistance testing by population sequencing before initiating ART [8], [9]. It is currently debated whether more sensitive techniques should be routinely applied to quantify minority drug-resistant HIV-1 variants, which remain undetected using population sequencing [10], [11]. The impact of pre-existing minority drug-resistant HIV-1 variants on treatment outcome was examined in numerous studies, however, there are still remaining open questions which drug resistance mutation is at which frequency, in which context, and at what point clinically relevant (reviewed in [12], [13], [14]).

The prevalence of minority drug-resistant HIV-1 variants in HIV-1 infected, treatment-naïve patients was measured in most studies at one time point closely before initiation of first-line ART. So far, kinetics of these virus populations in individual patients were examined exclusively in the context of ART. We have reported that minority drug-resistant HIV-1 variants can be rapidly selected in periods of inefficient ART [15], [16], [17]. Conversion to drug-sensitive viruses was found in patients interrupting treatment after virological failure, but minority drug-resistant HIV-1 variants may persist in these patients [18]. Furthermore, minority drug-resistant HIV-1 variants can emerge in patients undergoing multiple structured treatment interruption and can persist in off-ART periods [19].

Longitudinal analyses of natural fluctuations of minority drug-resistant HIV-1 variants have not been performed so far. Besides the evolutionary question about the stability of these variants within the virus population longitudinal analyses might also be of clinical importance. All studies on minority drug-resistant HIV-1 variants and their potential impact on ART have been performed retrospectively. Furthermore, most of the studies quantified minority drug-resistant HIV-1 variants in samples of the exact day or very shortly before initiation of ART. In practice or in prospective studies this is not an option. Thus, longitudinal investigations of fluctuations of minority drug-resistant HIV-1 variants in the absence of drug pressure will be useful to determine the clinical value of a certain minority drug-resistant HIV-1 variant at a certain time point. We have detected minority drug-resistant HIV-1 variants prior to initiation of early ART in acutely HIV-1 infected patients participating in the Zurich Primary HIV-1 Cohort (ZPHI) [20]. Here, we studied the reappearance and fate of these variants in patients who interrupted efficient ART. The K103N and M184V mutations were chosen, because they are commonly transmitted drug resistance mutations [6], [7].

## Methods

The protocol for this trial is available as supporting information; see Protocol S1.

### Ethics statement

The study protocol was approved by the Ethics committee of the University Hospital of Zurich.

### Study design and participants

The ZPHI study is an observational, open label, non-randomized, single center study (www.clinicaltrials.gov; ID:NCT00537966). During the course of this study, no deviations from the Trial Protocol occurred. Acute and recent HIV-1 infection is defined as described previously [20], [21]. Minority drug-resistant HIV-1 variants were measured in longitudinal samples of 17 patients during treatment interruption using allele-specific real-time PCR (AS-PCR). HIV-1 quantification, resistance testing, and AS-PCR for detection and quantification of minority drug-resistant HIV-1 variants harboring K103N and/or M184V mutations were performed as described previously [20]. Details of set-up of AS-PCR assays and data analysis are described elsewhere [19], [20]. Written informed consent was obtained from each patient prior to inclusion.

Treatment interruption is offered to patients to investigate the possible benefit of early ART to lower the viral set point and to gain more ART-free years. The necessity of life-long treatment, side effects, toxicity, and cost are considerable, thus, alternative strategies should be investigated.

## Results

Minority K103N and/or M184V HIV-1 variants were previously detected at the earliest available time point during acute or recent HIV-1 infection and before initiation of ART in 15 of 109 patients included in the ZPHI between March 2002 and December 2007 [20]. Six of these 15 patients were eligible for the current study where reappearance of minority HIV-1 variants was studied after intended interruption of suppressive ART (<40 HIV-1 RNA copies/ml plasma).

**Figure 1:**
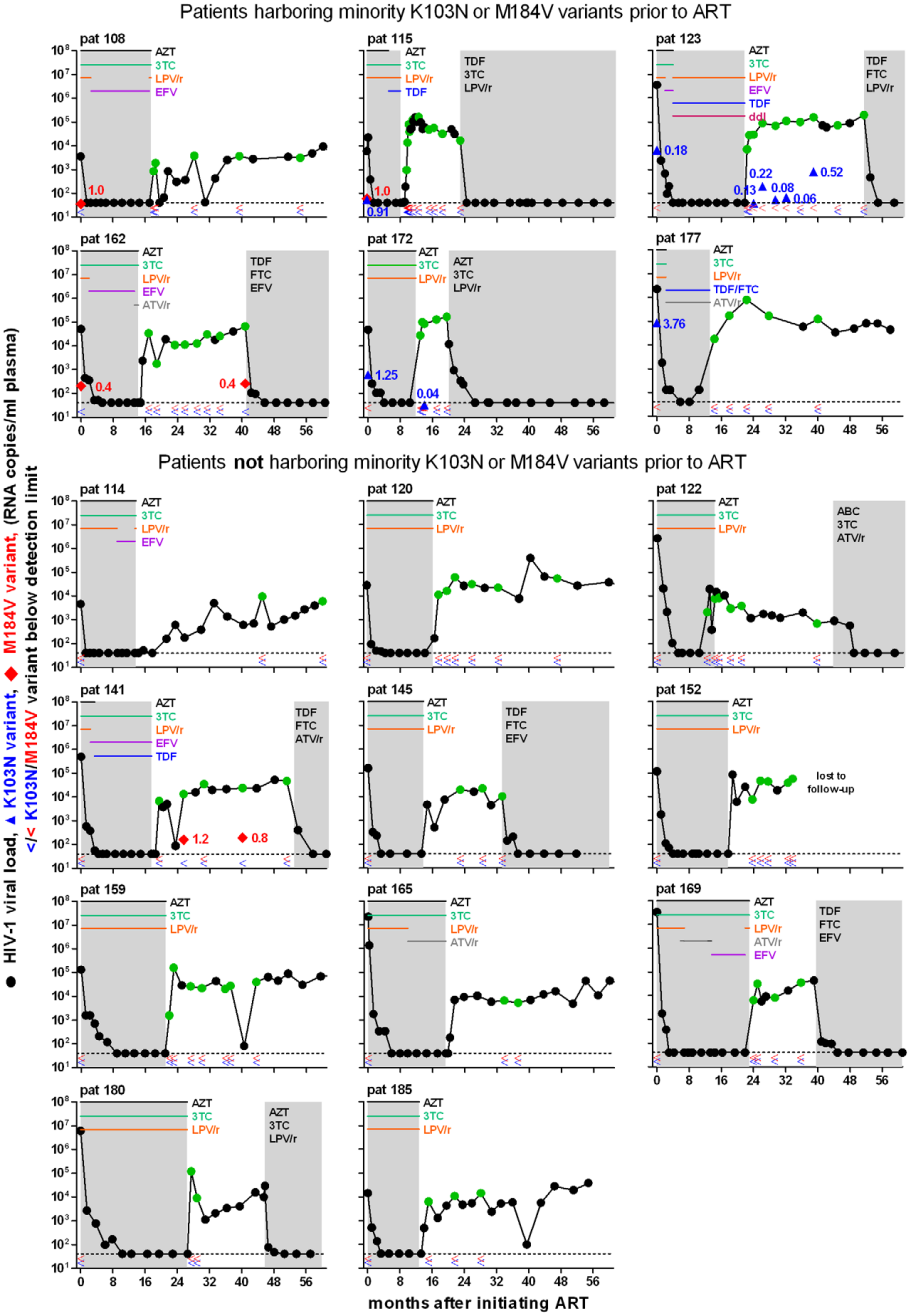
Kinetics of minority K103N and M184V HIV-1 variants in patients receiving early ART and undergoing intended treatment interruption. Plasma viral load was measured using the Cobas AmpliPrep/Cobas TaqMan HIV-1 Test (black and green circles, the latter represent the time points that were used for the quantification of minority drug-resistant HIV-1 variants). The limit of 40 HIV-1 RNA copies/ml plasma is shown in dashed lines. Periods of ART are depicted in grey shaded areas; the durations of specific drugs in the early ART regimens are indicated by differently coloured lines. In addition, the initial ART regimen after reintroduction of ART is depicted. Minority K103N (blue triangles) and M184V (red diamonds) HIV-1 variants were quantified by AS-PCR; percentages are given and were used to calculate the absolute amounts of these variants based on the viral load. Percent values below the assays discriminatory abilities (0.2% for M184V and 0.01% for K103N) or the individually calculated detection limits based on the viral load are shown by less-than signs. ART, antiretroviral therapy; AZT, zidovudine; 3TC, lamivudine; LPV, lopinavir; /r, ritonavir-boosted; EFV, efavirenz; TDF, tenofovir; ddI, didanosine; ATV, atazanavir; FTC, emtricitabine.

Nine patients were not eligible due to the following reasons: No start of ART during acute infection (n = 2), no treatment interruption (n = 5), and lost to follow-up (n = 2). Thus, six patients with and 11 patients without minority K103N and/or M184V HIV-1 variants prior to early ART were studied. The last-mentioned group of patients included all patients of the ZPHI who did not harbor minority drug-resistant HIV-1 variants at baseline, started early ART, interrupted treatment, had longitudinal plasma samples available with viral loads above 1,000 HIV-1 RNA copies/ml plasma, and were acutely HIV-1 infected within the same years the six patients with minority drug-resistant HIV-1 variants have acquired HIV-1 infection. These mutations are associated with resistance against non nucleos(t)ide analogue reverse transcriptase inhibitors (NNRTIs) and some NRTIs, respectively [22]. At baseline, the M184V mutation was detected in three and the K103N mutation in four patients at low frequencies; patient 115 harbored both variants (figure 1; table 1) [20]. All except one patient were acutely HIV-1 infected as defined by detailed criteria [21]. Patients 185 was recently infected with HIV-1, i.e., referred to our center more than 90 days after the estimated time point of infection, however, acute HIV-1 infection was documented (table 1). Drug resistance mutations defined as recommended by the International AIDS Society–USA Drug Resistance Mutations Group and the surveillance drug resistance mutations list [8], [23] were not detected by population sequencing in any patient. All patients were men who have sex with men and infected with HIV-1 subtype B between 2003 and 2005. All patients started early ART with zidovudine, lamivudine, and ritonavir-boosted lopinavir. ART was changed in nine patients due to gastrointestinal side effects, anemia, or simplification to once daily regimens (figure 1).

**Table 1:** Baseline characteristics of patients with primary HIV-1 infection.

							AS-PCR			
Patient ID	Sex	Route of infection	HIV-1 subtype	Year of primary HIV-1 infection	Estimated time point of infection (weeks)	HIV-1 RNA copies/ml plasma	K103N mean±SD (%)	M184V mean±SD (%)	Drug resistance mutations by GRT	1^st^ line treatment
**Patients harboring minority drug-resistant HIV-1 variants prior to early ART**										
108	M	MSM	B	2003	6.0	3,560	<d.l.	1.0±0.1	none	LPV/r, AZT, 3TC
115	M	MSM	B	2003	5.0	6,040	0.91±0.24	1.0±0.0	none	LPV/r, AZT, 3TC
123	M	MSM	B	2003	4.6	3,620,000	0.18±0.02	<d.l.	none	LPV/r, AZT, 3TC
162	M	MSM	B	2005	8.0	51,100	<d.l.	0.4±0.0	none	LPV/r, AZT, 3TC
172	M	MSM	B	2005	5.9	47,900	1.25±0.13	<d.l.	none	LPV/r, AZT, 3TC
177	M	MSM	B	2005	4.4	2,220,000	3.76±0.79	<d.l.	none	LPV/r, AZT, 3TC
**Patients not harboring minority drug-resistant HIV-1 variants prior to early ART**										
114	M	MSM	B	2003	5.7	4,610	<d.l.	<d.l.	none	LPV/r, AZT, 3TC
120	M	MSM	B	2003	10.1	27,800	<d.l.	<d.l.	none	LPV/r, AZT, 3TC
122	M	MSM	B	2003	7.9	2,610,000	<d.l.	<d.l.	none	LPV/r, AZT, 3TC
141	M	MSM	B	2004	5.0	495,500	<d.l.	<d.l.	none	LPV/r, AZT, 3TC
145	M	MSM	B	2004	5.4	157,000	<d.l.	<d.l.	none	LPV/r, AZT, 3TC
152	M	MSM	B	2004	11.7	114,000	<d.l.	<d.l.	none	LPV/r, AZT, 3TC
159	M	MSM	B	2004	6.9	132,000	<d.l.	<d.l.	none	LPV/r, AZT, 3TC
165	M	MSM	B	2005	2.1	22,200,000	<d.l.	<d.l.	none	LPV/r, AZT, 3TC
169	M	MSM	B	2005	1.7	33,150,000	<d.l.	<d.l.	none	LPV/r, AZT, 3TC
180	M	MSM	B	2005	3.6	6,050,000	<d.l.	<d.l.	none	LPV/r, AZT, 3TC
185	M	MSM	B	2005	18.4	14,400	<d.l.	<d.l.	none	LPV/r, AZT, 3TC

**Abbreviations**: AS-PCR, allele-specific real-time PCR; <d.l., below detection limit; GRT, genotypic resistance testing by population sequencing; M, male; MSM, men who have sex with men; LPV/r, lopinavir (boosted with ritonavir); AZT, zidovudine; 3TC, lamivudine.

Baseline characteristics were similar between patients with and without minority K103N and/or M184V HIV-1 variants prior to early ART in terms of viral load at baseline (median [range] HIV-1 RNA copies/ml plasma: 5.0×10^4^ [3.6×10^3^–3.6×10^6^] vs. 1.6×10^5^ [4.6×10^3^–3.3×10^7^]), time on ART before interruption (median [range] months: 13.7 [8.1–21.8] vs. 17.5 [10.4–26.6]), and duration of viral load <40 during ART (median [range] months: 7.3 [2.3–17.8] vs. 12.3 [5.3–18.9]).

In total, 43 and 45 longitudinal samples after treatment interruption were measured by AS-PCR for the mutations K103N and M184V in patients with and without these variants at low frequencies prior to early ART, respectively. The M184V mutation was detected three times in two patients (figure 1): In patient 162, this variant was present at baseline and reappeared once in the last of eight samples during treatment interruption. In patient 141, the M184V mutation was not present prior to early ART, but appeared in 2/5 samples after treatment interruption.

The K103N mutation was detected during treatment interruption in two patients harboring this mutation at low frequencies prior to early ART (figure 1). In patient 174, the K103N mutation reappeared in one of five time points. In patient 123, the K103N mutation reappeared 2.2 months after treatment interruption and fluctuated between 0.06 and 0.52% within the following 15 months in the absence of ART. During this time, the K103N mutation was once not detectable (individually calculated detection limit based on the viral load for this time point: 0.03%). This mutation disappeared and was not detectable in the two time points tested prior to reinitiation of ART. In addition, the first two time points after treatment interruption were also negative for the K103N mutation, however, the individually calculated detection limits were higher for these samples compared with the following time points due to lower viral loads (0.36% and 0.09%, respectively). The K103N mutation was neither detected during treatment interruption in the other two patients harboring this variant nor in the 13 patients not harboring minority K103N HIV-1 variants during primary HIV-1 infection. Reinitiation of ART was efficient to suppress viremia below detectable limits within a few weeks in all patients who reinitiated ART independent of the detection of minority K103N and/or M184V HIV-1 variants during treatment interruption (figure 1).

## Discussion

Reappearance and fluctuations of minority K103N and/or M184V HIV-1 variants was studied in patients initiating early ART during primary HIV-1 infection and undergoing intended treatment interruption of suppressive ART without any evidence for virological failure. Some patients harbored these minority drug-resistant HIV-1 variants prior to early ART, others did not. Different patterns could be observed for these mutants during treatment interruption: The M184V mutation was occasionally detected in two patients independent of its presence prior to early ART, which reflects probably sporadic and temporary appearance of this drug-resistant variant. On the other hand, the K103N mutation reappeared in two patients both harboring this minority variant prior to early ART. In one patient, minority K103N HIV-1 variants reappeared shortly after treatment interruption and persisted for more than 1.2 years in the absence of any selective pressure. Drug-related selection of K103N viruses is unlikely. One patient did never receive an NNRTI. The other patient only temporarily received efavirenz from months two to four, more than 1.5 years before treatment interruption. At month two, viral load was almost fully suppressed. We and others have shown that the selection of minority drug-resistant HIV-1 variants during the initial months of ART is unlikely when viremia rapidly decays [16], [20], [24]. Furthermore, the K103N mutation did not appear in any other of the five patients who switched from a ritonavir-boosted PI to efavirenz.

If selection was the cause for the appearance of drug-resistant variants after treatment interruption, it would have been anticipated that the M184V mutation rather than the K103N mutation is predominantly selected, because all patients received lamivudine, which can rapidly select M184V viruses [22]. However, selection of the M184V mutation during continuously fully suppressive ART in our patients is very unlikely. On the contrary, the random and seldom appearance of the M184V mutation in these patients suggests that viral replication is very low or most likely even completely inhibited during ART [25], [26], [27], [28], [29]. Differences in viral fitness could explain our observations. The presence of the M184V mutation alleviates virus fitness (reviewed in [30]) and these viruses are rapidly replaced by M184 wild-type viruses in the absence of selective pressure [31], thus, transient emergence, i.e., appearance and rapid disappearance, of HIV-1 variants with reduced viral fitness is probable during the time period of treatment interruption. On the contrary, the K103N mutation is not associated with a major impact on viral fitness [32], [33]. The reappearance and persistence of drug-resistant viruses without reduced replication capacities are likely after interruption of ART.

One limitation of our study is the small number of patients. We compensated this by testing many time points during treatment interruption. Thus, sporadic appearance of minority M184V HIV-1 variants could be demonstrated. This is in line with previous theoretical predictions [34]. K103N viruses were able to reappear and persist. That the K103N mutation also sporadically appears can not be excluded, however, this probably occurs less frequently compared to the M184V mutation. Furthermore, the discriminatory ability of the K103N AS-PCR assay is 20-fold more sensitive than the M184V AS-PCR, therefore, sporadic appearance of K103N viruses would have been more easily detectable, which was not observed.

Transmitted or once selected drug-resistant variants may persist as major virus population or as minority variants independent of the selective pressure by ART, which implies a threat to future antiretroviral regimens (reviewed in [35]). The prevalence of minority drug-resistant HIV-1 variants in treatment-naïve patients may reflect their transmission as major virus population and subsequent decay due to impaired viral fitness, their transmission and persistence as minority variant, or their sporadic appearance [34]. In some patients, the presence of minority drug-resistant HIV-1 variants can lead to rapid selection of these viruses and immediate therapy failure [17], [36]. An association between virological failure after temporary suppression of virus replication and the presence of minority drug-resistant HIV-1 variants has also been shown [37], [38], [39], [40], [41]. In contrast, other studies did not find such an association [20], [38], [40], [42], [43], [44], [45]. Because of such conflicting results and the complexity to correlate resistance data with clinical endpoints, clinical decision trees do not yet consider minority drug-resistant HIV-1 variants [8], [9]. However, certain minority drug-resistant HIV-1 variants may have an impact on ART and therefore it is important to study fluctuations of these populations in the absence of selective pressure.

In summary, we observed that the K103N mutation can reappear and persist at low frequencies in the absence of selective pressure after treatment interruption when it was already present as minority drug-resistant HIV-1 variant during primary HIV-1 infection. The M184V mutation does rather show sporadic appearance. Longitudinal studies of the kinetics of minority drug-resistant HIV-1 variants will provide further insights in the persistence and fluctuations of these viruses and will lead to better understanding of the clinical relevance of minority drug-resistant HIV-1 variants.

## Supporting Information

Protocol S1 Study protocol: INFZ-ZPHI-01.01 “Characterization of acute and recent HIV-1 infections in Zurich: a long-term observational study.”
(PDF)
