# Supplementary material for: Reappearance of Minority K103N HIV-1 Variants after Interruption of ART Initiated during Primary HIV-1 Infection
Source: PLoS One. 2011 Jul 6;6(7):e21734. doi: 10.1371/journal.pone.0021734 (PMC3130779; doi:10.1371/journal.pone.0021734)
Supplement: Protocol S1 — Study protocol: INFZ-ZPHI-01.01 “Characterization of acute and recent HIV-1 infections in Zurich: a long-term observational study.” (PDF) [file pone.0021734.s001.pdf]

STUDY PROTOCOL: INFZ-ZPHI-01.01

"Characterization of acute and recent HIV-1 infections in Zurich: a long-term observational study."

Principal Investigator:
Prof. Dr. med. Huldrych Günthard,
University Hospital Zürich, Division of Infectious Diseases and Hospital Epidemiology,
Rämistrasse 100,
8091 Zürich
Tel 41 1 255 34 50, Fax 41 1 255 32 91

1. Summary

Aim of the study: To describe the epidemiology, longitudinally follow, test the effect of early antiretroviral treatment and investigate early events of virus-host interactions in patients with documented acute or recent HIV-1 infection in Zurich.

Study design: This is an open label, non-randomized, observational, monocenter study at the University Hospital Zurich, Division of Infectious Diseases and Hospital Epidemiology. We aim at enrolling approximately 300 patients over a 10 year period. All patients who fulfill the inclusion criteria of a documented acute or recent HIV infection can participate in the study. Patients are offered early combination antiretroviral treatment (cART), if treatment start falls within 90 days after diagnosis of acute HIV-infection. After one year of suppressed HIV-plasma viremia (< 50 copies/ml) patients can chose to stop cART. Patients who have not chosen to undergo early-cART, respectively will stop cART after one year will be followed for a total of 5 years. Viral setpoints reached after treatment interruptions will be compared to historic controls and to the control group not having received cART during acute infection. A battery of virological and immunological assays will be performed on blood samples obtained to better understand early virus-host interactions, which are thought to play a key role in HIV-pathogenesis research.

Summary: In summary, this study will provide comprehensive knowledge on early HIV-infection with regard to epidemiology, impact of early-cART on the course of disease and forms the base for a variety of translational research projects addressing early key pathogenesis events between virus and host, relevant for the course of disease, for transmission, for development of vaccines and new treatment strategies.

2. Introduction

Background

Twenty years after emergence of the AIDS epidemic and a decade after introduction of highly successful combination antiretroviral therapy (cART) HIV-1 remains one of the three leading infectious diseases in the world causing a high burden of morbidity, mortality and costs [1, 2].

Despite an enormous body of knowledge accumulated, a number of key questions regarding HIV-pathogenesis remain unresolved. To date it is still not fully understood what factors determine the viral setpoint in a given individual. Even less is known about factors determining viral transmission. It has been recognized that longitudinal studies of HIV-infected patients early on after primary HIV infection (PHI) may be key to better understanding of HIV-pathogenesis [3-8]. Insights of early events between virus and host may potentially be crucial for development of vaccines, new drugs, microbiocides and new strategies optimizing long-term antiretroviral therapy. Despite 10 years of cART to date it remains unknown whether early-cART initiated during PHI can alter the course of disease in delaying disease progression [5, 9-14].

Thus, taken together there is a strong need for systematic longitudinal pathogenesis studies in humans infected with HIV-1 addressing transmission and early virus-host events.

Early antiretroviral treatment after primary HIV infection.

The optimal time to start antiretroviral therapy for HIV-infected patients remains unknown [15-18]. Even less clear is whether treatment of PHI early on is beneficial in terms of delaying disease progression. Recent work provided some rationale that early intervention during PHI might indeed be beneficial. Yerly et al. [19], demonstrated that viral rebound and setpoint upon interruption of cART in chronically infected patients is predicted by proviral DNA levels at time of treatment stop. Furthermore, Strain et al. [20] showed that reduction of the size of the latent reservoir is significantly more pronounced in early-cART treated PHI patients when compared to initiation of cART in chronic infection. Furthermore, this work also demonstrated that the decay of proviral HIV-DNA is similar to the decay of cell infectivity assessed by terminal dilution co-culture. In conjunction, one could hypothesize that a reduction of the latent reservoir indeed could translate into lowered viral setpoints and subsequent delay of disease progression. For this reason, proviral DNA as a valuable surrogate marker of the long lived cellular reservoir and potentially of the viral setpoint needs to be revisited in the context of primary HIV-infection. Furthermore, potentially other viral markers such as different HIV-RNA species [21-28] may be of additional value. A recent observational study suggested that early-cART treated PHI patients exhibited lower viral setpoints after stop of early-cART compared to controls when adjusted for baseline CD4 counts. Interestingly the CD4 count benefit observed, persisted for up to 72 weeks [9]. Another very recent study reported that a median duration of HAART of 1.1 years was associated with a persistent effect on viral load and CD4-T-cell count for up to 5 years after seroconversion [14]. In contrast to these results a short term treatment of 24 weeks did not show any benefit on viral setpoint and CD4 count [13]. In the near future, we mainly depend on observational studies because randomized clinical trials in this setting are not feasible [5].

Viral and host factors influence the HIV-1 viral setpoint

HIV infection is characterized by continuous viral replication at a high rate, which combined with the error rate of the reverse transcriptase [29, 30], frequent recombination [31, 32], and host selection pressure leads to a high genetic diversity in infected individuals [33-37]. However, the level of diversity between individual patients can vary considerably [36, 38-40]. After primary HIV infection each individual reaches a steady state level of plasma viral load which is referred to as viral setpoint. These setpoints between individuals can vary more than 1000 fold [41]. The switch in

coreceptor usage from CCR5 to CXCR4, which occurs in approximately 50% of patients, is associated with more vigorous viral replication in vivo exemplified by an increase in plasma viremia levels in HIV-infected individuals and more rapid disease progression [41-46]. Various viral and host properties may contribute to the observed diversity: these include differences in virulence, subtype, immunogenicity and replication capacity of the transmitted viruses, the quasispecies composition of the infecting inoculum (transmission of single versus multiple quasispecies), host genetic factors such as chemokine receptor polymorphisms, HLA types and gender differences [47-51]. HIV-specific CD4 and CD8 responses also contribute to control of viremia, however, optimal measurements for functional HIV-1 specific CD8 responses in humans have still to be determined. In particular, various recent studies in humans [10, 52-54] in contrast to earlier ones [55] were not able to link levels of HIV-specific CD8 responses with the viral setpoint. Neutralizing antibodies arising within weeks to months after PHI are also thought to contribute to the viral setpoint. Evidence, however, is mostly indirect by escape of neutralization responses and viral evolution of the *env* gene most likely driven by neutralizing antibodies directed against env [35, 37, 56]. Recently, we have shown that higher neutralizing antibody titers against autologous viruses indeed did correlate with viral setpoint reached in patients undergoing structured treatment interruptions [38]. More convincingly, first direct evidence for neutralizing activity *in vivo* was demonstrated by our passive immunization study, where a cocktail of three broadly neutralizing antibodies was administered to patients with suppressed viremia. Upon stop of cART, viral rebound could be delayed significantly, demonstrating that neutralizing antibodies indeed can have a direct effect on viral replication *in vivo*[57, 58] [59].

In addition to viral factors and immune responses it has been well described, that a variety of host genetic factors are influencing HIV-1 disease progression in a negative or positive way. Polymorphisms at the following genotypic locations have been found to influence disease progression: CCR5 delta 32, CCR5 G-2455A, CCR2 V64I, RANTES G-403A, RANTES C-28G, MIP-1a T113C, SDF-1 3'A. Moreover, a variety of HLA alleles have been found to accelerate AIDS (HLA-A, B, C homozygosity [60, 61], HLA-B*35 [62, 63], HLA-A*01-B*08-DRB1*03 [64, 65] and others have been demonstrated to delay disease progression such as HLA-B*57 and HLA*B27 [66]. Of interest one HLA association was even shown to correlate with maintenance of viral suppression in patients treated early after seroconversion; HLA-DRB1*13-DQB*6 [67]. Recently, a whole genome association study of major determinants for host control revealed three new relevant polymorphisms which can explain approx. 15% of the variation among viral setpoints in untreated asymptomatic patients [68]. For these reasons, it is evident that predictors of the viral setpoint cannot be studied anymore without also assessing these important factors – at least in part - in modulating the course of HIV-1 disease. Nevertheless, it has been shown many times that effects of these host genetic traits are subtle within single individuals and large patient cohorts are normally needed to demonstrate such effects. Thus, for our project we do not focus exclusively on host genetics, but rather will perform these analysis to exclude a potential bias as we have done so before [24, 69].

Taken together, prerequisites to comprehensively study viral and host factors are well defined patient groups that are followed longitudinally.

Current status of treatment interruptions in HIV-infected patients.

Originally, structured treatment interruptions were studied systematically for three reasons: Firstly, to test the autovaccination hypothesis, secondly, to reduce toxicity, and thirdly, to reduce exposure to antiretroviral drugs and therefore reduce costs. The autovaccination hypothesis has been rejected in chronically infected as well as in patients infected during primary HIV infection, meaning that short term-treatment interruptions, despite the fact that they could induce HIV-specific T-helper, -cytotoxic CD8 and humoral immune responses were not able to sustainably lower viral setpoints [10, 53, 54, 70-72]. CD4-guided treatment interruptions in lower CD4 strata were associated with increased frequencies of HIV-, non-HIV-related morbidity and non-HIV related mortality in two

large trials [73, 74]. However, increased morbidity and mortality were not seen in higher CD4-strata [75-77]. No data of structured treatment interruptions in large clinical trials performed in patients with primary HIV-infections are available to date. However, the fact that morbidity and mortality seen in the large trials were dependent at least in part on the CD4 stratum in which the cycling took place suggests that the risk for interruption of early ART introduced during PHI is minimal because patients with early-cART in most cases do reach normal or almost normal CD4 counts within the first year of treatment. Thus, taken together, the risk of harming patients who stop early PHI treatment is not existent or very low, given that high CD4 counts are reached after early PHI treatment, and that negative treatment interruption effects seen in chronic HIV-infection were very rare in general and CD4-dependen at least to some extent.

Current status of the Zurich PHV infection cohort

The Zurich PHI study has already been started at the beginning of 2002. First, patients were enrolled in the Merck study 520, Protocol No 013-00 (EK 851). At that timepoint we have already applied for a study extension in addition to the Merck protocol and this was accepted under the EK 851 and later on 1086). From our own research initiative, several manuscripts have already been published over the last 3 years:

1. Trkola A, Kuster H, Rusert P, Joos B, Fischer M, Leemann C, Manrique A, Huber M, Rehr M, Oxenius A, Weber R, Stiegler G, Vcelar B, Katinger H, Aceto L, Günthard HF. Delay of HIV-1 rebound after cessation of antiretroviral therapy through passive transfer of human neutralizing antibodies. Nature Medicine. 2005:11:615-622.
2. Rusert P, Kuster H, Joos B, Misselwitz B, Gujer C, Leemann C, Fischer M, Stiegler G, Katinger H, Olson WC, Weber R, Aceto L, Günthard HF, Trkola A. Virus isolates during acute and chronic Human Immunodeficiency Virus Type 1 infection show distinct patterns of sensitivity to entry inhibitors. Journal of Virology. 2005;79:8454-8469.
3. Aceto L, Karrer U, Grube C, Oberholzer R, Hasse B, Presterl E, Böni J, Kuster H, Trkola A, Weber R, Günthard HF. Die Akute HIV Infektion in Zürich: 2002 – 2004. [Primary HIV-1 Infection in Zurich: 2002-2004] PRAXIS (Schweizerische Rundschau für Medizin) 2005;94:1199-1205.
4. Joos B, Trkola A, Aceto L, Fischer M, Vcelar B, Stiegler G, Katinger H, Kuster H, Günthard HF. Multiple dose pharmakokinetics of combined anti-HIV-1 human monoclonal antibodies 2G12, 4E10 and 2F5 administered intravenously to HIV-infected patients. Antimicrobial Agents and Chemotherapy. 2006;50:1773-1779.
5. Huber M, Fischer M, Misselwitz B, Manrique A, Kuster H, Niederöst B, Von Wyl V, Weber R, Günthard HF and Trkola A. Antibody and complement mediated lysis activity contributes to viremia control in the acute phase of HIV-1 infection. PLOS Medicine 2006; 2006;3:2078-2093. (e441)
6. Manrique A, Rusert P, Joos B, Fischer M, Kuster H, Leemann C, Niederöst B, Weber R, Stiegler G, Katinger H, Günthard HF, Trkola A. In vivo and in vitro escape from neutralizing antibodies 2G12, 2F5 and 4E10. Journal of Virology 2007:81:8793-8808.

This study so far has been highly successful and for this reason it was decided to continue with our own protocol after Merck has stopped to enrol patients. To date we have enrolled more than 140 patients with documented primary HIV infection within the first 5 years. An interim analysis of 62 patients [78] focusing on clinical aspects like delay to diagnose HIV on first physician contact due to difficulty to recognize the "acute retroviral syndrome" (ARS), symptoms and signs, laboratory abnormalities, primary resistance and mode of transmission revealed that clinical characteristics of the Zurich PHI cohort are comparable with other published PHI studies [79-81]. Approximately 90% of patients so far have chosen to undergo early-cART. To this end 50 patients have stopped antiretroviral treatment. Primary resistance has only been detected in 4% so far and approx. 70% were infected with a subtype B strain. Criteria for diagnosis of PHI are: 1) acute retroviral syndrome [78] and negative or indeterminate westernblot in the presence of a positive p24Ag and/or detectable plasma HIV-1 RNA. 2) documented seroconversion with or without symptoms within 90 (acute infection) or 180 days (recent infection). 3) possible ARS, positive westernblot and detectable HIV-RNA, and a negative HIV-gp120 avidity assay [82, 83] or a detuned assay [84] (recent infection). Overall, 73 patients (63%) had a negative or indeterminate westernblot, respectively 97 patients could unambiguously be identified to be acutely infected. Thus, the Zurich PHI cohort is well characterized and suitable for the work proposed here.

3. Study objectives

Primary study objectives:

- To enrol and longitudinally follow patients with documented primary HIV infection referred to the Division of Infectious Diseases, University Hospital Zurich. (either defined as acute or recent infection).

Secondary study objectives:

- To evaluate the effect of early-cART on the viral setpoint
- To determine viral factors, potentially relevant for HIV-1 pathogenesis: a) Transmission of drug resistance, b) cellular levels of proviral DNA, c) levels of different cell associated HIV-RNA species, d) viral diversity, e) replication capacity, f) isolation of replication competent virus
- To determine HIV specific immune responses: a) kinetics of anti-HIV-1 specific humoral immune responses, b) kinetics of HIV-specific cellular immune responses, c) investigation of factors associated with the innate immune system.
- To determine genetic traits known to be associated with differential progression in natural HIV-infection, such as (HLA typing, chemokine receptor polymorphisms, cellular factors, potentially new genes of interest).
- To search for viral and immunological factors which are associated with HIV-1 transmission.

4. Patient population

As per 30 July 2007 148 patients were enrolled under the former protocols approved by the Ethics committee (No. EK 852 and EK 1086). All patients enrolled previously will be asked to sign the informed consent of the current project (INFZ-ZPHI 01.01, Version 1) retrospectively. We expect to enroll another 150 patients by the end of 2011. This is a realistic scenario assuming the same rate of new HIV-1 infections as we have observed over the last 5 years.

Patient enrollment:

Patients with documented primary HIV-1 infection who agree to participate in the current study will receive the following routine screening at baseline: total blood count, routine chemistry laboratory (e.g. creatinine, liver enzymes, lipid profile, c-reactive protein, serum-glucose), HIV-1 RNA, p24Ag, HIV-genotypic drug resistance test, Western-blot, (HIV-screening if still necessary), CD4 count, and further laboratory testing belonging to the standard procedure for patients presenting with new HIV-infection (Hepatitis, A, B, C serologies, CMV-, syphilis-, toxoplasmosis- serologies etc.). All these tests are covered by regular health insurance because they reflect standard of care.

Inclusion Criteria:

- Patients of 18 or more years who present with a documented primary HIV-infection defined as:

A) **acute HIV-1 infection**, defined as:

- acute retroviral syndrome [78] (ARS) and negative or indeterminate Westernblot in the presence of a positive p24 Ag and/or detectable plasma HIV-1 RNA
- documented seroconversion with or without symptoms within 90 days.

or

B) **recent HIV-1 infection**, defined as:

- possible ARS, positive Westernblot and detectable HIV-RNA, and a negative HIV-gp120 avidity [82, 83], respectively detuned assay [84].
- documented acute HIV-1 infection, however, referral to our center more than 90 days after presumed date of infection.

Exclusion criteria:

- Hemoglobin < 10 g/dl (men) and < 9 g/dl (women) at the time of enrollment.

5. Study site

This is an open label, non-randomized monocenter, observational study, conducted at the

University Hospital Zurich
Division of Infectious Diseases and Hospital Epidemiology
Rämistrasse 100
8091 Zürich

6. Intervention

Patients with intervention:

Intervention a):
Early combined antiretroviral treatment (early-cART): All patients with documented acute HIV-1 infection, presenting within 90 days after presentation of ARS or documented seroconversion will be offered early combination antiretroviral treatment with a standard firstline cART regimen as recommended in treatment guidelines [85] containing drugs that are approved by Swissmedic. Treatment will be continued until at least 12 months complete HIV-RNA suppression in the plasma has been achieved (HIV-plasma RNA < 50 copies/ml for at least 12 months (see Figure 1).

Intervention b):
Interruption of early-cART: After one year of fully suppressive early-cART patients can stop cART and then will be switched to the observational study arm (see Figure 1).

Patients without intervention:

All patients with documented acute HIV-1 infection who do not want to have early-cART, respectively patients with documented recent infection will be enrolled in the observational study group only (see Figure 1).

7. Drug regimes selected for early-cART

Initial treatment consists of a ritonavir-boosted protease inhibitor (PI) combined with two nucleoside reverse transcriptase inhibitors (nRTI) (e.g. lopinavir/ritonavir: 2 x per day 200/50 mg in combination with zidovudin/lamivudin: 2 x per day, 300/150 mg). This is a well established, potent

drug combination with excellent efficacy as a first line treatment. This treatment is chosen because the boosted protease inhibitor has a high genetic barrier, which is particularly important because NNRTI resistance mutations transmitted, may hamper the whole NNRTI based first line treatment from the beginning (von Wyl et al, Archives of Internal Medicine, 2007, in press). If side effects or toxicity do arise, respectively results from the resistance test recommends a different drug regimen, different early-cART regimens are chosen. In this case the following alternative boosted-PI regimens will be chosen (atazanavir/ritonavir: once daily 300/100 mg, darunavir/ritonavir: 2 x per day 600mg/100mg). If a boosted-PI regimen is not possible, a non-nucleoside-reverse-transcriptase inhibitor (NNRTI) based regimen (e.g. efavirenz: once daily 600mg or nevirapin: 200mg 2 x per day, or once daily 400mg) is also possible. As alternative NRTI regimens, abacavir and lamivudin: once daily 600/300mg, or tenofovir and emtricitabin: once daily 300mg/200mg will be used.

8. Safety of cART

Safety of cART will be monitored the same way as this is done in clinical routine: week 2, 4, 8, 12 and thereafter every three months. In women who undergo early-cART and are pregnant a drug regimen will be chosen, which excludes efavirenz.

9. Study flow chart

a) The diagram below shows a schematic outline of the study:

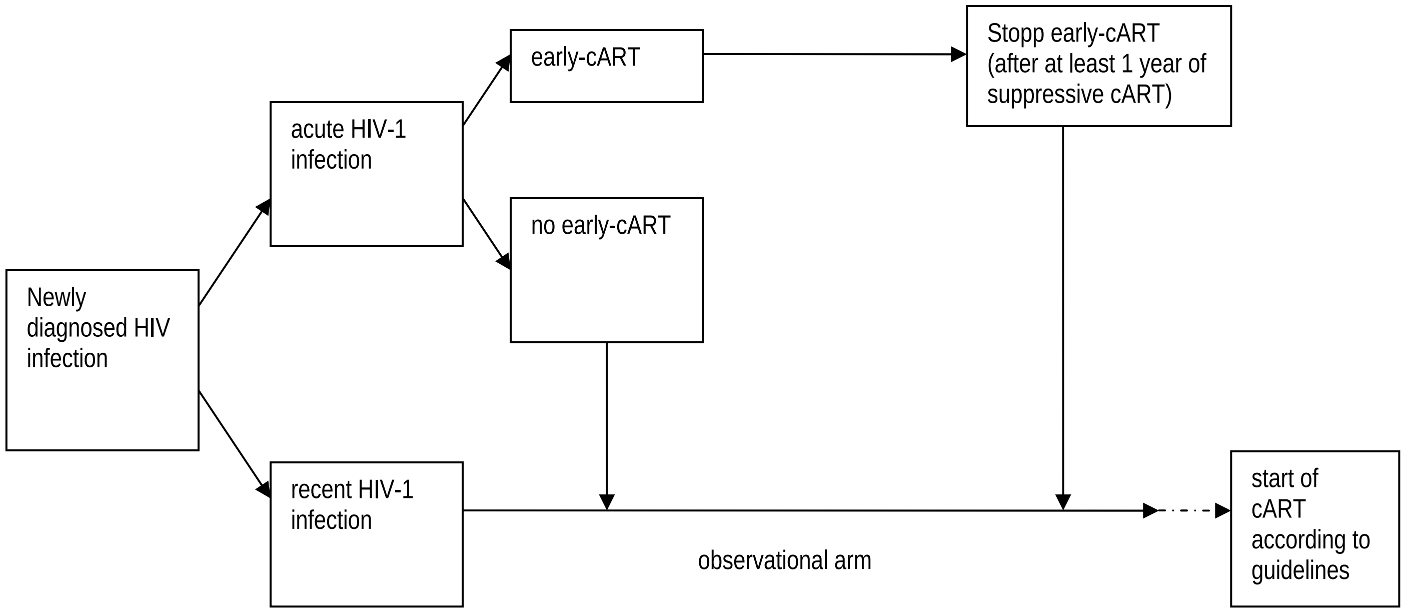

b) Under **Appendix I, III, III** sequential blood drawing is shown schematically in three tables:

Appendix I (page 15-16): This table depicts patient visits and planed amounts of blood that will be drawn from patients who will undergo early-cART. Furthermore, in the right column a synopsis of the total amount of blood per timepoint (blood for clinical routine purposes and study blood) is shown.

Appendix II (page 17-18)**:** This table shows patient visits and planed amounts of blood that will be drawn from patients who will interrupt their early-cART treatment after at least one year of successful suppression (plasma HIV-RNA <50 RNA copies/ml for at least 12 months). Furthermore, in the right column a synopsis of the total amount of blood per timepoint (blood for clinical routine purposes and study blood) is shown.

Appendix III (page 19-20)**:** This table shows patient visits and planed amounts of blood that will be drawn from patients who have not chosen to start early-cART treatment or who have presented with "recent infection". Furthermore, in the right column a synopsis of the total amount of blood per timepoint (blood for clinical routine purposes and study blood) is shown.

c) Specimen Collection and Processing

Blood specimens will be obtained according to the tables of appendix I – III.
EDTA-Blood from all time points will be separated into cells and plasma according to standard procedures. Cellular material is divided into aliquots that are stored either in DMSO medium in liquid nitrogen to yield viable cells for immunological analysis, or stored at –70°C as dry cell

pellets to provide material for genetic analysis and viral load measurements. All plasma samples are stored at –70°C.

11. Endpoints and statistical analysis

Analysis of the parameters described under secondary study objectives will be performed upon availability of data. Exact description of these research projects are not feasible within this study protocol. Briefly, the following analyses are planned:

- Viral setpoints of patients having stopped early-cART will be compared with viral setpoints from patients with primary HIV infection without early-cART and with viral setpoints from patients with documented time of seroconversion enrolled in the Swiss HIV cohort study (historic controls). A first interim analysis will be performed in 2009.
- Moreover, the cART free interval between patients underoing early-cART (from timepoint of stopping their first cART to timepoint of initiation of second cART according to current guidelines) and patients without early-cART (timepoint of initiation of cART according to current guidelines) will be determined.
- In addition, analysis of parameters described under secondary study objectives will be performed after the research data has been generated. In general, these parameters will be measured longitudinally, and kinetics of these parameters will be determined and compared between groups with and without early-cART.

Standard statistical methods will be used for these analyses. Details of the planned analyses have been described in Swiss National Science Foundation research proposals:
"Humoral immunity to HIV-1: Analysis of the function of antibody responses as correlate and surrogate marker of viremia control", SNF Nr. 3100A0-103748 *"Factors associated with viral control after primary HIV-1 infection and viral determinants associated with HIV-1 transmission.", SNF No. 324700-116035* (Principle Investigator: H. Günthard).

12. Time frame

The current study is the successor of two previous studies: Merck study 520 (EK 851) and the extension of that study (EK 1086). Patient enrollment for these two studies has been conducted between January 2002 – End of August 2007.

We will enroll patients under this new protocol as soon as it is accepted by the ethics committee. We expect to enroll patients from September 2007 – end of 2011. Laboratory investigations and the observational period will continue until 2016.

13. Laboratory investigations

a) "Standard of Care" clinical laboratory investigations: Laboratory investigations in the context of clinical care will be carried out by the various diagnostic laboratories of the University Hospital and the Univerity of Zurich.

b) **"Research laboratory investigations":** Isolation of autologous virus, humoral immune responses including neutralizing antibody escapes, antibody dependent cellular cytotoxicity (ADCC), innate immune responses, (complement lysis, natural killer cell activity), isolation and

studies of B-Zell function, determination of proviral DNA and of different HIV-RNA species, determination of viral diversity, envelope sequencing and viral replication capacity assays will all be performed in the HIV research laboratory of the Division of Infectious Diseases and Hospital Epidemiology in collaboration with Prof. Dr. A. Trkola and PD Dr. M. Fischer.

The following laboratory research will be conducted in collaboration with:

- HIV-specific cellular immune responses (Prof. A. Oxenius, ETH Zurich)
- Determination of HIV-drug resistant minority quasispecies (PD Dr. K. Metzner, University of Erlangen).

14. Ethics and Trial registration

Informed consent.

Informed consent from each subject participating in the trial will be obtained before any trial specific procedures are performed on the subject. The aims, methods, benefits, and potential hazards of the trial will be explained to each participant before signing the informed consent.
It will be made clear that each subject can withdraw their consent at any time and for any reason, without incurring any penalty or withholding of treatment on the part of the investigator.
Signed informed consent forms will be kept on file by the investigator and a copy of it will be given to the patient upon request.

Patients who have already been enrolled in the EK protocol No 851 and 1086 will be consented again under this new protocol.

This trial will be registered in the trial registry of the University Hospital Zurich and in the international trial registry “clinical.trials.gov” (www.clinical.trials.gov).

15. Safety features and insurance

Safety evaluations.

- Vital signs will be monitored at baseline (body temperature, pulse and blood pressure).
- Physical examination will be performed at baseline and later on if needed.
- Complete Blood Count (CBC) including white blood cell differential, and platelet count: at baseline
- Chemistry including AST, ALT, bilirubin, creatinin, at baseline
- Urine analysis (Uristix and sediment): at baseline and later on if needed.
- CD4 count: at baseline
- HIV-plasma RNA: at baseline
- genotypic HIV drug resistance testing: at baseline

For patients who will undergo early antiretroviral treatment safety laboratory (chemistry and CBC will be repeated at week 2, 4, 8, 12 and then every three months thereafter.

Viral load and CD4 monitoring occurs monthly until VL is undetectable and thereafter every three months.

For patients not undergoing early-cART, plasma HIV-RNA and CD4 count will be repeated every 3 months and CBC and chemistry will be repeated every 6 months as it is done in standard clinical care.

If the study participants encounter damage caused by the present study, such damage is covered by the insurance policy of the University Hospital Zurich.

16. Feasibility

The fact that 148 patients have already been recruited (January 1, 2002 - July 30, 2007) under the previous protocols (EK 851 and EK 1086) demonstrates that the study is feasible. The infrastructure and know-how in the clinic and research laboratories is in place. Thus, the capacity to conduct the study is available. Furthermore, all collaborations are working and fully active.

17. Finances

The current study is sponsored by the Division of Infectious Diseases and Hospital Epidemiology, University Hospital Zurich. Research expenses are covered by a variety of project specific separate research grants (Swiss National Science Foundation and private foundations).

18. Investigators

Principal investigator: Prof. Dr. med. Huldrych Günthard
Co-Investigator: Prof. Dr. med. Rainer Weber

Both at the University Hospital Zurich, Division of Infectious Diseases and Hospital Epidemiology, Zurich, Switzerland

19. Publications

If, at the end or during the trial, publications or presentations of the results of this trial are planned then all parties involved in the respective research work have the right to review the manuscript and abstracts before their submission. However, decision for submission of a manuscript or abstract is made by the principal investigator.

Zürich, 7 August 2007 Principal Investigator

Prof. Dr. med. H. Günthard

20. References

1. Fauci AS. The global challenge of infectious diseases: the evolving role of the National Institutes of Health in basic and clinical research. Nat Immunol 2005;6:743-7
2. Fauci AS. Twenty-five years of HIV/AIDS. Science 2006;313:409
3. Derdeyn CA, Decker JM, Bibollet-Ruche F, et al. Envelope-constrained neutralization-sensitive HIV-1 after heterosexual transmission. Science 2004;303:2019-22
4. Frost SD, Liu Y, Pond SL, et al. Characterization of human immunodeficiency virus type 1 (HIV-1) envelope variation and neutralizing antibody responses during transmission of HIV-1 subtype B. J Virol 2005;79:6523-7
5. Kinloch-de Loes S. Treatment of Acute HIV-1 Infection: Is It Coming of Age? J Infect Dis 2006;194:721-4
6. Mattapallil JJ, Douek DC, Hill B, Nishimura Y, Martin M and Roederer M. Massive infection and loss of memory CD4+ T cells in multiple tissues during acute SIV infection. Nature 2005;434:1093-7
7. Smith DE, Walker BD, Cooper DA, Rosenberg ES and Kaldor JM. Is antiretroviral treatment of primary HIV infection clinically justified on the basis of current evidence? Aids 2004;18:709-18
8. Watson A, McClure J, Ranchalis J, et al. Early postinfection antiviral treatment reduces viral load and prevents CD4+ cell decline in HIV type 2-infected macaques. AIDS Res Hum Retroviruses 1997;13:1375-81
9. Hecht FM, Wang L, Collier A, et al. A Multicenter Observational Study of the Potential Benefits of Initiating Combination Antiretroviral Therapy during Acute HIV Infection. J Infect Dis 2006;194:725-33
10. Kaufmann DE, Lichterfeld M, Altfeld M, et al. Limited durability of viral control following treated acute HIV infection. PLoS Med 2004;1:e36
11. Kinloch-de Loes S, Hoen B, Smith DE, et al. Impact of therapeutic immunization on HIV-1 viremia after discontinuation of antiretroviral therapy initiated during acute infection. J Infect Dis 2005;192:607-17
12. Rosenberg ES, Altfeld M, Poon SH, et al. Immune control of HIV-1 after early treatment of acute infection. Nature 2000;407:523-6
13. Streeck H, Jessen H, Alter G, et al. Immunological and Virological Impact of Highly Active Antiretroviral Therapy Initiated during Acute HIV-1 Infection. J Infect Dis 2006;194:734-9
14. Touloumi GP, N.; Porter, K.; on behalf of the CASCADE Collaboration. Long term virological and immunological effects of HAART initiated in the first 12 months after HIV-1 seroconversion. In: 3rd International AIDS Socienty Conference on HIV Pathogenesis and Treatmetnt. Rio de Janeiro, 2005
15. Phillips AN, Lepri AC, Lampe F, Johnson M and Sabin CA. When should antiretroviral therapy be started for HIV infection? Interpreting the evidence from observational studies. Aids 2003;17:1863-9
16. Sterling TR, Chaisson RE, Keruly J and Moore RD. Improved outcomes with earlier initiation of highly active antiretroviral therapy among human immunodeficiency virus-infected patients who achieve durable virologic suppression: longer follow-up of an observational cohort study. J Infect Dis 2003;188:1659-65
17. Walensky RP, Goldie SJ, Sax PE, et al. Treatment for primary HIV infection: projecting outcomes of immediate, interrupted, or delayed therapy. J Acquir Immune Defic Syndr 2002;31:27-37
18. Wang C, Vlahov D, Galai N, et al. Mortality in HIV-seropositive versus -seronegative persons in the era of highly active antiretroviral therapy: implications for when to initiate therapy. J Infect Dis 2004;190:1046-54
19. Yerly S, Gunthard HF, Fagard C, et al. Proviral HIV-DNA predicts viral rebound and viral setpoint after structured treatment interruptions. Aids 2004;18:1951-3
20. Strain MC, Little SJ, Daar ES, et al. Effect of treatment, during primary infection, on establishment and clearance of cellular reservoirs of HIV-1. J Infect Dis 2005;191:1410-8
21. Fischer M, Kaiser, P., Baumann, D., Hafner, R., Schneider, C., Bonhoeffer, S., Weber, R., Günthard, H. Decay Kinetics of PBMC with Different HIV-RNA Expression Patterns Measured at the Single-cell Level Using Limiting Dilution Combined with Patient Mached Real-time PCR. In: 12th Conference on Retroviruses and Opportunistic Infections, February, 22-25. Boston, 2005
22. Fischer M, Gunthard HF, Opravil M, et al. Residual HIV-RNA levels persist for up to 2.5 years in peripheral blood mononuclear cells of patients on potent antiretroviral therapy. AIDS Res Hum Retroviruses 2000;16:1135-40
23. Fischer M, Hafner R, Schneider C, et al. HIV RNA in plasma rebounds within days during structured treatment interruptions. Aids 2003;17:195-9
24. Fischer M, Joos B, Hirschel B, Bleiber G, Weber R and Gunthard HF. Cellular viral rebound after cessation of potent antiretroviral therapy predicted by levels of multiply spliced HIV-1 RNA encoding nef. J Infect Dis 2004;190:1979-88
25. Fischer M, Joos B, Wong JK, et al. Attenuated and nonproductive viral transcription in the lymphatic tissue of HIV-1-infected patients receiving potent antiretroviral therapy. J Infect Dis 2004;189:273-85
26. Fischer M, Trkola A, Joos B, et al. Shifts in cell-associated HIV-1 RNA but not in episomal HIV-1 DNA correlate with new cycles of HIV-1 infection in vivo. Antivir Ther 2003;8:97-104
27. Fischer M, Wong JK, Russenberger D, et al. Residual cell-associated unspliced HIV-1 RNA in peripheral blood of patients on potent antiretroviral therapy represents intracellular transcripts. Antivir Ther 2002;7:91-103

28. Kaiser P, Joos B, Niederost B, Weber R, Gunthard HF and Fischer M. Productive Human Immunodeficiency Virus 1 Infection in peripheral Blood Predominantly Takes Place in CD4/CD8 double negative T Lymphocytes. J Virol 2007
29. Coffin JM. HIV viral dynamics. Aids 1996;10 Suppl 3:S75-84
30. McCutchan FE. Understanding the genetic diversity of HIV-1. Aids 2000;14 Suppl 3:S31-44
31. Fang G, Weiser B, Kuiken C, et al. Recombination following superinfection by HIV-1. Aids 2004;18:153-9
32. Shriner D, Rodrigo AG, Nickle DC and Mullins JI. Pervasive genomic recombination of HIV-1 in vivo. Genetics 2004;167:1573-83
33. Leslie AJ, Pfafferott KJ, Chetty P, et al. HIV evolution: CTL escape mutation and reversion after transmission. Nat Med 2004;10:282-9
34. Price DA, Goulder PJ, Klenerman P, et al. Positive selection of HIV-1 cytotoxic T lymphocyte escape variants during primary infection. Proc Natl Acad Sci U S A 1997;94:1890-5
35. Richman DD, Wrin T, Little SJ and Petropoulos CJ. Rapid evolution of the neutralizing antibody response to HIV type 1 infection. Proc Natl Acad Sci U S A 2003;100:4144-9
36. Shankarappa R, Margolick JB, Gange SJ, et al. Consistent viral evolutionary changes associated with the progression of human immunodeficiency virus type 1 infection. J Virol 1999;73:10489-502
37. Wei X, Decker JM, Wang S, et al. Antibody neutralization and escape by HIV-1. Nature 2003;422:307-12
38. Joos B, Trkola A, Fischer M, et al. Low human immunodeficiency virus envelope diversity correlates with low in vitro replication capacity and predicts spontaneous control of plasma viremia after treatment interruptions. J Virol 2005;79:9026-37
39. Strain MC, Letendre S, Pillai SK, et al. Genetic composition of human immunodeficiency virus type 1 in cerebrospinal fluid and blood without treatment and during failing antiretroviral therapy. J Virol 2005;79:1772-88
40. Joos B, Fischer M, Schweizer A, et al. Positive In Vivo Selection of the HIV-1 Envelope Protein gp120 Occurs at Surface-Exposed Regions. J Infect Dis 2007;196:313-20
41. Piatak M, Jr., Saag MS, Yang LC, et al. High levels of HIV-1 in plasma during all stages of infection determined by competitive PCR. Science 1993;259:1749-54
42. Blaak H, Ran LJ, Rientsma R and Schuitemaker H. Susceptibility of in vitro stimulated PBMC to infection with NSI HIV-1 is associated with levels of CCR5 expression and beta-chemokine production. Virology 2000;267:237-46
43. Blaak H, van't Wout AB, Brouwer M, et al. Infectious cellular load in human immunodeficiency virus type 1 (HIV-1)-infected individuals and susceptibility of peripheral blood mononuclear cells from their exposed partners to non-syncytium-inducing HIV-1 as major determinants for HIV-1 transmission in homosexual couples. J Virol 1998;72:218-24
44. Connor RI, Sheridan KE, Ceradini D, Choe S and Landau NR. Change in coreceptor use coreceptor use correlates with disease progression in HIV-1--infected individuals. J Exp Med 1997;185:621-8
45. Kwa D, Vingerhoed J, Boeser B and Schuitemaker H. Increased in vitro cytopathicity of CC chemokine receptor 5-restricted human immunodeficiency virus type 1 primary isolates correlates with a progressive clinical course of infection. J Infect Dis 2003;187:1397-403
46. Schuitemaker H, Kootstra NA, Groenink M, De Goede RE, Miedema F and Tersmette M. Differential tropism of clinical HIV-1 isolates for primary monocytes and promonocytic cell lines. AIDS Res Hum Retroviruses 1992;8:1679-82
47. Sagar M, Kirkegaard E, Long EM, et al. Human immunodeficiency virus type 1 (HIV-1) diversity at time of infection is not restricted to certain risk groups or specific HIV-1 subtypes. J Virol 2004;78:7279-83
48. Sagar M, Lavreys L, Baeten JM, et al. Infection with multiple human immunodeficiency virus type 1 variants is associated with faster disease progression. J Virol 2003;77:12921-6
49. Scherer A, Frater J, Oxenius A, et al. Quantifiable cytotoxic T lymphocyte responses and HLA-related risk of progression to AIDS. Proc Natl Acad Sci U S A 2004;101:12266-70
50. Smith MW, Dean M, Carrington M, et al. Contrasting genetic influence of CCR2 and CCR5 variants on HIV-1 infection and disease progression. Hemophilia Growth and Development Study (HGDS), Multicenter AIDS Cohort Study (MACS), Multicenter Hemophilia Cohort Study (MHCS), San Francisco City Cohort (SFCC), ALIVE Study. Science 1997;277:959-65
51. Trachtenberg E, Korber B, Sollars C, et al. Advantage of rare HLA supertype in HIV disease progression. Nat Med 2003;9:928-35
52. Betts MR, Ambrozak DR, Douek DC, et al. Analysis of total human immunodeficiency virus (HIV)-specific CD4(+) and CD8(+) T-cell responses: relationship to viral load in untreated HIV infection. J Virol 2001;75:11983-91
53. Oxenius A, McLean AR, Fischer M, et al. Human immunodeficiency virus-specific CD8(+) T-cell responses do not predict viral growth and clearance rates during structured intermittent antiretroviral therapy. J Virol 2002;76:10169-76
54. Oxenius A, Price DA, Gunthard HF, et al. Stimulation of HIV-specific cellular immunity by structured treatment interruption fails to enhance viral control in chronic HIV infection. Proc Natl Acad Sci U S A 2002;99:13747-52
55. Ogg GS, Jin X, Bonhoeffer S, et al. Quantitation of HIV-1-specific cytotoxic T lymphocytes and plasma load of viral RNA. Science 1998;279:2103-6
56. Frost SD, Gunthard HF, Wong JK, Havlir D, Richman DD and Leigh Brown AJ. Evidence for positive selection driving the evolution of HIV-1 env under potent antiviral therapy. Virology 2001;284:250-8

57. Joos B, Trkola A, Kuster H, et al. Long-term multiple-dose pharmacokinetics of human monoclonal antibodies (MAbs) against human immunodeficiency virus type 1 envelope gp120 (MAb 2G12) and gp41 (MAbs 4E10 and 2F5). Antimicrob Agents Chemother 2006;50:1773-9
58. Trkola A, Kuster H, Rusert P, et al. Delay of HIV-1 rebound after cessation of antiretroviral therapy through passive transfer of human neutralizing antibodies. Nat Med 2005;11:615-22
59. Manrique A, Rusert P, Joos B, et al. In Vivo and In Vitro Escape from Neutralizing Antibodies 2G12, 2F5, and 4E10. J Virol 2007;81:8793-808
60. Carrington M, Nelson GW, Martin MP, et al. HLA and HIV-1: heterozygote advantage and B*35-Cw*04 disadvantage. Science 1999;283:1748-52
61. Tang J, Costello C, Keet IP, et al. HLA class I homozygosity accelerates disease progression in human immunodeficiency virus type 1 infection. AIDS Res Hum Retroviruses 1999;15:317-24
62. Gao X, Nelson GW, Karacki P, et al. Effect of a single amino acid change in MHC class I molecules on the rate of progression to AIDS. N Engl J Med 2001;344:1668-75
63. Itescu S, Mathur-Wagh U, Skovron ML, et al. HLA-B35 is associated with accelerated progression to AIDS. J Acquir Immune Defic Syndr 1992;5:37-45
64. Flores-Villanueva PO, Hendel H, Caillat-Zucman S, et al. Associations of MHC ancestral haplotypes with resistance/susceptibility to AIDS disease development. J Immunol 2003;170:1925-9
65. Kaslow RA, Duquesnoy R, VanRaden M, et al. A1, Cw7, B8, DR3 HLA antigen combination associated with rapid decline of T-helper lymphocytes in HIV-1 infection. A report from the Multicenter AIDS Cohort Study. Lancet 1990;335:927-30
66. Kaslow RA, Carrington M, Apple R, et al. Influence of combinations of human major histocompatibility complex genes on the course of HIV-1 infection. Nat Med 1996;2:405-11
67. Malhotra U, Holte S, Dutta S, et al. Role for HLA class II molecules in HIV-1 suppression and cellular immunity following antiretroviral treatment. J Clin Invest 2001;107:505-17
68. Fellay J, Shianna KV, Ge D, et al. A Whole-Genome Association Study of Major Determinants for Host Control of HIV-1. Science 2007
69. Trkola A, Kuster H, Leemann C, et al. Human immunodeficiency virus type 1 fitness is a determining factor in viral rebound and set point in chronic infection. J Virol 2003;77:13146-55
70. Fagard C, Oxenius A, Gunthard H, et al. A prospective trial of structured treatment interruptions in human immunodeficiency virus infection. Arch Intern Med 2003;163:1220-6
71. Trkola A, Kuster H, Leemann C, et al. Humoral immunity to HIV-1: kinetics of antibody responses in chronic infection reflects capacity of immune system to improve viral set point. Blood 2004;104:1784-92
72. Kaufmann DE, Rosenberg ES. The value of preserving HIV-specific immune responses. J HIV Ther 2003;8:19-25
73. El-Sadr WM, Lundgren JD, Neaton JD, et al. CD4+ count-guided interruption of antiretroviral treatment. N Engl J Med 2006;355:2283-96
74. Danel C, Moh R, Minga A, et al. CD4-guided structured antiretroviral treatment interruption strategy in HIV-infected adults in west Africa (Trivacan ANRS 1269 trial): a randomised trial. Lancet 2006;367:1981-9
75. Ananworanich J, Gayet-Ageron A, Le Braz M, et al. CD4-guided scheduled treatment interruptions compared with continuous therapy for patients infected with HIV-1: results of the Staccato randomised trial. Lancet 2006;368:459-65
76. Ananworanich J, Hirschel B. Intermittent therapy for the treatment of chronic HIV infection. Aids 2007;21:123-34
77. Ruiz L, Paredes R, Gomez G, et al. Antiretroviral therapy interruption guided by CD4 cell counts and plasma HIV-1 RNA levels in chronically HIV-1-infected patients. Aids 2007;21:169-78
78. Aceto L, Karrer U, Grube C, et al. [Primary HIV-1 infection in Zurich: 2002-2004]. Schweiz Rundsch Med Prax 2005;94:1199-205
79. Daar ES, Little S, Pitt J, et al. Diagnosis of primary HIV-1 infection. Los Angeles County Primary HIV Infection Recruitment Network. Ann Intern Med 2001;134:25-9
80. Kahn JO, Walker BD. Acute human immunodeficiency virus type 1 infection. N Engl J Med 1998;339:33-9
81. Schacker T, Collier AC, Hughes J, Shea T and Corey L. Clinical and epidemiologic features of primary HIV infection. Ann Intern Med 1996;125:257-64
82. Binley JM, Arshad H, Fouts TR and Moore JP. An investigation of the high-avidity antibody response to glycoprotein 120 of human immunodeficiency virus type 1. AIDS Res Hum Retroviruses 1997;13:1007-15
83. Rusert P, Kuster H, Joos B, et al. Virus isolates during acute and chronic human immunodeficiency virus type 1 infection show distinct patterns of sensitivity to entry inhibitors. J Virol 2005;79:8454-69
84. Janssen RS, Satten GA, Stramer SL, et al. New testing strategy to detect early HIV-1 infection for use in incidence estimates and for clinical and prevention purposes. Jama 1998;280:42-8
85. Hammer SM, Saag MS, Schechter M, et al. Treatment for adult HIV infection: 2006 recommendations of the International AIDS Society-USA panel. Jama 2006;296:827-43

Zürich PHI - Studie (INFZ-ZPHI-01) - Synopsis Blutentnahmen für Patienten mit Frühtherapie

SHCS Nr. .... **geb. Datum ....** **Initialen ....**

Name .... **Vorname** ....

Datum	Studien Phase	Visiten Nr.	Woche	Visiten Zürich	Routine Labor USZ	Blutabnahme Anzahl Röhrchen Zürich EDTA 10ml für Fried.	Total/ ACD 10ml Herbert	Blutmenge ml Studie	Blutmenge ml Routine	Blutmenge ml Total	Synopsis der Blutmenge geordnet nach Studienphasen	ml
					AKI,NZR							
	**Screening** * (1. Visite)	0	minus x	x	(RES,VL,CD4) (Häm, Chemie) (W-Blot, p24)	4	4	64	60	124	**Total Blutmenge in 3 Monaten**	**444**
....	**Studieneintritt***	1	0	x	CD4/RNA	10	0	80	20	100		
....	**Induktionsphase**	2	4	x	RNA	4	0	32	20	52		
....	**Induktionsphase**	3	8	x	CD4/RNA	8	0	64	20	84		
....	**Induktionsphase**	4	12	x	CD4/RNA	8	0	64	20	84		
....	**Beobachtungsph.**	5	16	x	CD4/RNA	5	0	40	20	60	**Total Blutmenge in 9 Monate**	**368**
....	**Beobachtungsph.**	6	24	x	CD4/RNA	7	0	56	20	76		
....	**Beobachtungsph.**	7	32	x	CD4/RNA	5	0	40	32	72		
....	**Beobachtungsph.**	8	40	x	CD4/RNA	5	0	40	20	60		
....	**Beobachtungsph**	9 (Ende Jahr 1)	48	x	CD4/RNA	10	0	80	20	100		
....	**Beobachtungsph.**	10	68	x	CD4/RNA	5	0	40	32	72	**Total Blutmenge Jahr 2**	**244**
....	**Beobachtungsph.**	11	84	x	CD4/RNA	5	0	40	20	60		
....	**Beobachtungsph.**	12 (Ende Jahr 2)	100	x	CD4/RNA	8	0	64	48	112		

				Visiten	Routine Labor	Blutabnahme Anzahl Röhrchen		Blutmenge ml	Blutmenge ml	Blutmenge ml	Synopsis der Blutmenge geordnet nach Studienphasen	
**Datum**	**Studien Phase**	**Visiten Nr.**	**Woche**	**Zürich**	USZ	**Zürich EDTA 10ml**	**Total/ ACD 10ml**	Studie	Routine	**Total**		**ml**
					AKI,NZR	**für Fried.**	**Herbert**					
....	**Beobachtungsph.**	13	116	x	CD4/RNA	5	0	40	28	68	**Tot. Blutmenge Jahr 3**	**300**
....	**Beobachtungsph.**	14	132	x	CD4/RNA	5	0	40	20	60		
....	**Beobachtungsph.**	15	148	x	CD4/RNA	5	0	40	48	88		
....	**Beobachtungsph.**	16 (Ende Jahr 3)	164	x	CD4/RNA	8	0	64	20	84		
....	**Beobachtungsph.**	17	180	x	CD4/RNA	5	0	40	32	72	**Total Blutmenge Jahr 4**	**244**
....	**Beobachtungsph.**	18	196	x	CD4/RNA	5	0	40	20	60		
....	**Beobachtungsph.**	19 (Ende Jahr 4)	212	x	CD4/RNA	8	0	64	48	112		
....	**Beobachtungsph.**	20	228	x	CD4/RNA	5	0	40	32	72	**Total Blutmenge Jahr 5**	**244**
....	**Beobachtungsph.**	21	244	x	CD4/RNA	5	0	40	20	60		
....	**Beobachtungsph.**	22 (Ende Jahr 5)	260	x	CD4/RNA	8	0	64	48	112		

Blutvolumen für verschiedene Routine Blutentnahmen:

HIV RNA: 6ml, CD4 Zellen: 3 ml, Blutbild: 3 ml, Routine - Chemie: 3 ml, Serothek HIV Kohortenstudie: 12 ml, Zytothek HIV Kohortenstudie: 16 ml
HIV-Resistenztest: 10 ml, Westerblot: 8 ml, p24 Ag: 8 ml

*** Visite bevor antiretrovirale Therapie begonnen wird** (Resistenz, SHCS, andere baseline Laboruntersuchungen (Chemie, Hämat, Serologien etc))
Falls Diagnose schon klar ist von auswärts her, kann Screening Visite und Studieneintritts-Visite zusammengefasst werden (dann 10 EDTA für Friederike und 4 ACD für Herbert)

Appendix II:

Zürich PHI - Studie (INFZ-ZPHI-01) - Synopsis Blutentnahmen nach Stop der Frühtherapie

SHCS Nr. .... *geb. Datum ....* **Initialen ....**

Name .... **Vorname** ....

Datum	Studien Phase / für wen	Visiten Nr.	Monat	Woche	Visiten Zürich	Routine Labor USZ AKI	Blutmenge Anzahl Röhrchen Zürich EDTA 10ml für F. Burgener	Blutmenge ml Studie	Blutmenge ml Routine	Blutmenge ml Total	Synopsis der Blutmenge geordnet nach Studienphasen	ml
....	stop ART	0	0	0	x	(VL,CD4)	10	80	20	**100**	Total Blutmenge für 1. Jahr Nachbeobachtungszeit	**640**
....	Nachbeob-Phase	1		2	wiss	0	3	24	0	**24**		
....	Nachbeob-Phase	2	1	4	x	(VL)	5	40	0	**40**		
....	Nachbeob-Phase	3		6	wiss	0	3	24	0	**24**		
....	Nachbeob-Phase	4	2	8	x	(VL, CD4)	10	80	0	**80**		
....	Nachbeob-Phase	5	3	12	x	(VL)	3	24	20	**44**		
....	Nachbeob-Phase	6	4	16	x	(VL,CD4)	10	80	0	**80**		
....	Nachbeob-Phase	7	5	20	x	(VL)	3	24	0	**24**		
....	Nachbeob-Phase	8	6	24	wiss	0	5	40	32	**72**		
....	Nachbeob-Phase	9	7	28	x	(VL,CD4)	3	24	0	**24**		
....	Nachbeob-Phase	10	9	36	x	(VL,CD4)	5	40	0	**40**		
....	Nachbeob-Phase	11	12	52	x	(VL,CD4)	5	40	48	**88**		

Datum	Studien Phase	Visiten Nr.	Monat	Woche	Visiten Zürich	Routine Labor USZ AKI	Blutmenge Anzahl Röhrchen Zürich EDTA 10ml für F. Burgener	Blutmenge ml Studie	Blutmenge ml Routine	Blutmenge ml Total	Synopsis der Blutmenge geordnet nach Studienphasen	ml
	für wen											
....	**Nachbeob-Phase**	12	16	64	x	(VL,CD4)	3	24	0	**24**	**Total Blutmenge für 2. Jahr**	**208**
....	**Nachbeob-Phase**	13	19	76	x	(VL,CD4)	5	40	32	**72**		
....	**Nachbeob-Phase**	14	22	88	x	(VL,CD4)	3	24	0	**24**		
....	**Nachbeob-Phase**	15	25	100	x	(VL,CD4)	5	40	48	**88**		
....	**Nachbeob-Phase**	16	28	112	x	(VL,CD4)	3	24	0	**24**	**Total Blutmenge für 3. Jahr**	**208**
....	**Nachbeob-Phase**	17	31	124	x	(VL,CD4)	5	40	32	**72**		
....	**Nachbeob-Phase**	18	34	136	x	(VL,CD4)	3	24	0	**24**		
....	**Nachbeob-Phase**	19	37	148	x	(VL,CD4)	5	40	48	**88**		
....	**Nachbeob-Phase**	20	40	160	x	(VL,CD4)	3	24	0	**24**	**Total Blutmenge für 4. Jahr**	**208**
....	**Nachbeob-Phase**	21	43	172	x	(VL,CD4)	5	40	32	**72**		
....	**Nachbeob-Phase**	22	46	184	x	(VL,CD4)	3	24	0	**24**		
....	**Nachbeob-Phase**	23	49	196	x	(VL,CD4)	5	40	48	**88**		
....	**Nachbeob-Phase**	24	52	208	x	(VL,CD4)	3	24	0	**24**	**Total Blutmenge für 5. Jahr**	**248**
....	**Nachbeob-Phase**	25	55	220	x	(VL,CD4)	5	40	0	**40**		
....	**Nachbeob-Phase**	26	58	232	x	(VL,CD4)	3	24	32	**56**		
....	**Nachbeob-Phase**	27	61	244	x	(VL,CD4)	5	40	0	**40**		
....	**Nachbeob-Phase**	28	64	256	x	(VL,CD4)	5	40	48	**88**		

Zürich PHI - Studie (INFZ-ZPHI-01) - Synopsis Blutentnahmen Primo-Studie ohne Früh-Therapie

**SHCS Nr. ....**	***geb. Datum ....***	**Initialen ....**

Name .... **Vorname** ....

					**Visiten**	**Routine Labor**	**Blutmenge** Anzahl Röhrchen		**Blutmenge** ml	**Blutmenge ml**	**Blutmenge ml**	**Synopsis der Blutmenge geordnet nach Studienphasen**	
**Datum**	**Studien Phase**	**Visiten Nr.**	**Monat**	**Woche**	**Zürich**	**USZ**	**Zürich EDTA 10ml**	**Zürich ACD 10ml**	Studie	Routine	**Total**		**ml**
	**für wen**					AKI,NZR	**für F. Burgener**	**Herbert**					
	**Screening**	**0**	**0**	**minus x**	**x**	(RES,VL,CD4) (Häm, Chemie) (W-Blot, p24)	4	4	64	60	**124**	**Total Blutmenge für 1. Jahr Studie ohne Therapie**	**636**
....	**Eintritt in Studie**	1	0	0	x	(VL, CD4)	10	0	80	20	**100**		
....	**keine Therapie**	2		2	x	(VL)	3	0	24	0	**24**		
....	**keine Therapie**	3	1	4	x	(VL, CD4)	5	0	40	0	**40**		
....	**keine Therapie**	4		6	**wiss**	0	3	0	24	0	**24**		
....	**keine Therapie**	5	2	8	x	(VL, CD4)	10	0	80	0	**80**		
....	**keine Therapie**	6	3	12	x	(VL, CD4)	3	0	24	20	**44**		
....	**keine Therapie**	7	6	24	x	(VL, CD4)	10	0	40	32	**72**		
....	**keine Therapie**	8	9	36	x	(VL,CD4)	5	0	40	0	**40**		
....	**keine Therapie**	9	12	52	x	(VL,CD4)	10	0	40	48	**88**		
....	**keine Therapie**	10	16	64	x	(VL,CD4)	3	0	24	0	**24**	**Total Blutmenge für 2. Jahr**	**208**
....	**keine Therapie**	11	19	76	x	(VL,CD4)	5	0	40	32	**72**		
....	**keine Therapie**	12	22	88	x	(VL,CD4)	3	0	24	0	**24**		
....	**keine Therapie**	13	25	100	x	(VL,CD4)	5	0	40	48	**88**		

					**Visiten**	**Routine Labor**	**Blutmenge** Anzahl Röhrchen		**Blutmenge** ml	**Blutmenge ml**	**Blutmenge ml**	**Synopsis der Blutmenge geordnet nach Studienphasen**	
**Datum**	**Studien Phase**	**Visiten Nr.**	**Monat**	**Woche**	**Zürich**	**USZ**	**Zürich EDTA 10ml**	**Zürich ACD 10ml**	Studie	Routine	**Total**		**ml**
	**für wen**					AKI,NZR	**für F. Burgener**	**Herbert**					
....	**keine Therapie**	14	28	112	x	(VL,CD4)	3	0	24	0	**24**	**Total Blutmenge für 3. Jahr**	**208**
....	**keine Therapie**	15	31	124	x	(VL,CD4)	5	0	40	32	**72**		
....	**keine Therapie**	16	34	136	x	(VL,CD4)	3	0	24	0	**24**		
....	**keine Therapie**	17	37	148	x	(VL,CD4)	5	0	40	48	**88**		
....	**keine Therapie**	18	40	160	x	(VL,CD4)	3	0	24	0	**24**	**Total Blutmenge für 4. Jahr**	**208**
....	**keine Therapie**	19	43	172	x	(VL,CD4)	5	0	40	32	**72**		
....	**keine Therapie**	20	46	184	x	(VL,CD4)	3	0	24	0	**24**		
....	**keine Therapie**	21	49	196	x	(VL,CD4)	5	0	40	48	**88**		
....	**keine Therapie**	22	52	208	x	(VL,CD4)	3	0	24	0	**24**	**Total Blutmenge für 5. Jahr**	**248**
....	**keine Therapie**	23	55	220	x	(VL,CD4)	5	0	40	0	**40**		
....	**keine Therapie**	24	58	232	x	(VL,CD4)	3	0	24	32	**56**		
....	**keine Therapie**	25	61	244	x	(VL,CD4)	5	0	40	0	**40**		
....	**keine Therapie**	26	64	256	x	(VL,CD4)	5	0	40	48	**88**		
